# Topology-Dependent Antifreeze Properties of Biomimetic Linear and Star-Shaped Peptoids

**DOI:** 10.3390/biomimetics10060368

**Published:** 2025-06-04

**Authors:** Lei Feng, Liugen Xu, Junhao Wen, Minghai Zhao, Amjad Ali, Naushad Ahmad, Jianwei Lu, Li Guo

**Affiliations:** 1 School of Materials Sciences and Engineering, Jiangsu University, Zhenjiang 212013, China; 2 College of Materials Science and Engineering, Nanjing Forestry University, Nanjing 210037, China; 3 Department of Chemistry, College of Science, King Saud University, Riyadh 11451, Saudi Arabia

**Keywords:** star-shaped peptoid, cryopreservation, antifreeze properties

## Abstract

Developing safe and efficient cryoprotectants is critical for effective cryopreservation in biomedical applications. Inspired by natural antifreeze proteins (AFPs), a series of linear and star-shaped peptoids featuring isopropanol side chains to mimic the amphiphilic characteristics of threonine were prepared. The effects of chain length and molecular topology on antifreeze properties were systematically investigated. Both ice recrystallization inhibition (IRI) and ice crystal growth suppression improved with increasing chain length, and star-shaped peptoids exhibited superior performance. Notably, the star-shaped peptoid S-(A_6_)_3_ demonstrated excellent antifreeze activity and low cytotoxicity, highlighting its promise as a novel, non-toxic alternative to conventional cryoprotectants like DMSO. These findings provide valuable insight into the structure-property relationship of peptoids for cryopreservation applications.

## 1. Introduction

Currently, cryopreservation is the most effective method for the long-term preservation of living cells [1,2,3]. However, the growth of ice crystals at low temperatures can cause cellular dehydration, contraction, or intracellular freezing necrosis. Cells are subjected to ice crystal damage during both cryopreservation and thawing processes, and this damage is irreversible [4,5]. Effectively reducing ice crystal damage is crucial for cryopreservation of living cells, tissues, and organs. The introduction of cryoprotectants is considered an effective way to address ice crystal damage.

With the continuous development of cryobiology, researchers have discovered natural antifreeze proteins (AFPs) and various types of synthetic cryoprotectants [6,7,8,9,10]. All of them have the ability to lower the freezing point, slow down ice crystal growth, and prevent ice recrystallization. However, AFPs can cause rejection reactions, leading to biological hazards, and their extraction is extremely costly, which limits their further application. Therefore, synthetic cryoprotectants have attracted attention from researchers. Commonly used synthetic cryoprotectants currently include dimethyl sulfoxide (DMSO), glycerol, polyethylene glycol (PEG), and polyvinyl alcohol (PVA) [11,12]. However, they have the disadvantages of relatively poor cryoprotective effects and certain toxicity, which prevent their wide applications in fields such as biomedicine and food processing that require high safety standards [13]. To make progress in the field of cryopreservation, the development of cryoprotectants with high efficiency and safety is urgently needed but still challenging.

Peptoids, also known as N-substituted polyglycines, are a type of peptidomimetic biomaterial. The side-chain substituents of peptoids are located on the N atoms, and the main chain lacks chiral centers and amide hydrogen bonds [14,15]. Therefore, peptoids exhibit better solubility and thermal processability. Additionally, peptoids possess several advantages, such as good biocompatibility, resistance to enzymatic degradation, and low cytotoxicity, which makes them suitable for a wide range of applications in biomedicine, biosensors, chemical catalysis, and nanomaterials [16,17,18,19,20,21,22]. The research by Mia L. Huang revealed the potential of peptoids as ice crystallization modifiers, which could be used as antifreeze agents in food production and biomedicine [23]. Our research group has already investigated that peptoid oligomers with different substituents possess good antifreeze properties, suggesting the potential of peptoids as cryoprotectants [24,25]. However, the effect of peptoid structure, especially topological structure, on their antifreeze activities is still underexplored. It is known that modifying the structure of peptoids can adjust steric hindrance and electronic effects, influencing their spatial conformation and thereby changing their performance. We reasoned that linear and star-shaped peptoids should have different antifreeze properties. Unlike linear peptoids, the synthesis of star-shaped peptoids does not have many reports. Peschko et al. designed star-shaped peptoids using a grafting method, which requires multiple complex modifications [26]. However, the traditional solid-phase submonomer synthesis method can also be used to synthesize star-shaped peptoids. Jin et al. proposed an effective strategy for the synthesis of three-armed star-shaped peptoids [27]. By introducing a primary amine containing two amino groups as a central pivot during the single-chain synthesis process, the peptoid can be split into two branches, forming a three-armed star-shaped structure. This method provided a convenient way to prepare different star-shaped peptoids and made it possible to study their antifreeze properties.

In this study, by mimicking the amphiphilic functional groups present in threonine residues of natural AFPs, the isopropyl alcohol group having both hydrophobic methyl and hydrophilic hydroxyl groups was designed as a peptoid side chain. A series of linear and star-shaped peptoids with different chain lengths and structures were synthesized to investigate the effects of structures on antifreeze properties. It is hoped that more efficient cryoprotectants can be developed through continuous exploration of the structure and properties of peptoids.

## 2. Materials and Methods

### 2.1. Materials

Isopropylamine, triethylamine, dichloromethane, anhydrous magnesium sulfate, methanol, N,N-dimethylformamide (DMF), 4-methylpiperidine, bromoacetic acid, and trifluoroacetic acid (TFA) were purchased from Macklin (Shanghai, China). Rink Amide-AM resin (0.3~0.8 mmolg^−1^), dimethyldichlorosilane, N,N′-diisopropylcarbodiimide (DIC), acetonitrile, and triisopropylsilane (TIS) were purchased from Aladdin (Shanghai, China). Trimethylchlorosilane and deuterated chloroform were purchased from J&K Scientific (Shanghai, China). Mouse fibroblasts (L929) were purchased from MeiSen Cell Biotechnology LTD. RPMI 1640 medium, trypsin, and fetal bovine serum were purchased from Bio-Channel Biotechnology. Cell Counting Kit-8 (CCK-8) was purchased from Adamas. Except for dichloromethane, which was purified through a solvent purification system before use, all other solvents and reagents were directly used without any further purification.

### 2.2. Protection of the Hydroxyl Group in Isopropanolamine

In a round-bottom flask, 300 mL of anhydrous dichloromethane, 7.8 mL of isopropanolamine, and 21.1 mL of triethylamine were added; a magnetic stir bar was placed, and the mouth of the flask was sealed with a rubber stopper. After stirring in an ice bath for 10 minutes, 15.5 mL of trimethylchlorosilane was slowly added using a syringe. The reaction mixture was stirred in an ice bath for 20 min and then stirred at room temperature for 16 h. The resulting solution was washed with 150 mL of distilled water and dried with anhydrous magnesium sulfate. After filtration, the solvent was evaporated to afford product as a transparent liquid (13.8 g, 92.4% yield).

### 2.3. Synthesis of Peptoids

A pre-prepared silanization solution (C_2_H_6_Cl_2_Si/DCM, *v*/*v* = 1:19) was added into the synthesis tube and stood for 30 min before removing it. The tube was sequentially washed with DCM and CH_3_OH and then dried. A total of 600 mg amide resin was added to the synthesis tube and swollen with 12 mL N,N′-dimethylformamide (DMF). After filtration, 6 mL of 4-methylpiperidine/DMF (*v*/*v* = 1:4) solution was added twice, stirring with nitrogen bubbling for 2 min and 12 min, respectively. After filtration, the resin was washed with DMF 5 times. A total of 6 mL of 0.6 M bromoacetic acid/DMF solution and 520 μL of N,N′-diisopropylcarbodiimide (DIC) were added and stirred for 10 min until the acylation reaction was completed. After filtration, the resin was washed with DMF 5 times. A total of 6 mL of 1 M primary amine/DMF solution was added and stirred for 10 min for the substitution reaction. After filtration, the resin was washed with DMF 5 times. The acylation and primary amine substitution steps were repeated to obtain linear peptoids or the first arm of star-shaped peptoids. 1,3-propanediamine was used as the center of star-shaped peptoids. Firstly, 6 mL of 0.6 M bromoacetic acid/DMF solution and 520 μL of DIC were added and reacted for 10 min. Then, 6 mL of 0.6 M 1,3-propanediamine/DMF solution was added and reacted for 30 min for the substitution reaction. When synthesizing the subsequent two chains, 6 mL of 1.2 M bromoacetic acid/DMF solution and 1040 μL of DIC were added and reacted for 15 min for the acylation reaction. A total of 12 mL of 1 M primary amine/DMF solution was added and reacted for 30 min for the substitution reaction. After each reaction step, the resin was washed with DMF 5 times. After the last reaction step, the resin was further washed with DCM 3 times. After drying at room temperature, the resulting resin was stored at −18 °C. Peptoids were cleaved from the resin by a reaction with a solution of trifluoroacetic acid, water, and triisopropylsilane (*v*/*v*/*v* = 95/2.5/2.5). Crude peptoid products were obtained by evaporating volatiles and purified by preparative high-performance liquid chromatography (HPLC) and then lyophilization.

### 2.4. Ice Recrystallization Experiment

The ice recrystallization experiment employed the Splat Cooling Assay (SCA) method to assess the ice recrystallization inhibition (IRI) activity of the samples. Purified peptoid samples were thoroughly dissolved in PBS buffer solution to prepare sample solutions at three different concentrations: 1, 5, and 10 mg·mL^−1^. A glass slide was placed flat on a low-temperature workstation. The temperature was lowered to −60 °C at a rate of 20 °C per minute. Within 3 min, the sample solution was dropped from a height of 1.5 m above the low-temperature workstation onto the center of the glass slide and rapidly formed an ultra-thin solid ice layer. The temperature was then raised to −6 °C and maintained for 30 min to allow the ice layer to recrystallize and grow. The size of the ice crystals during the recrystallization process was monitored “in situ and in real time” under a microscope. Over the next 20 min, ten micrographs of the sample ice crystals from different positions were captured by a high-speed camera. The size of the ten largest ice grains in each image, defined by their two largest orthogonal dimensions, was measured using the Nano Measurer software (version 1.2). The average of all the data obtained was taken as the maximum large grain size (MLGS) of the sample ice crystals. The IRI activity of different peptoids was characterized by the relative percentage of the MLGS of the peptoid samples to that of PBS. Each sample was tested for at least three parallel experiments.

### 2.5. Nanoliter Osmometry Experiment

The nanoliter osmometry experiment was conducted to examine the morphology and the growth rate of ice crystals in the samples. Purified peptoid samples were thoroughly dissolved in pure water to prepare sample solutions at three different concentrations: 1, 5, and 10 mg·mL^−1^. Using a micropipette, the solutions were injected into a six-well plate filled with immersion oil, and the temperature was rapidly cooled to −20 °C until freezing occurred. The nanoliter osmometer was adjusted to slowly raise the temperature to the melting point, melting the ice until only a stable single ice crystal remained. Then, the temperature was adjusted to a supercooled state, and when the ice crystal just began to grow, a high-speed camera was used to record the ice crystal growth process. The ice crystal growth rate was calculated as the increase in the size of a single ice crystal divided by the time taken during the growth process. Each sample was tested for at least three parallel experiments.

### 2.6. Cytotoxicity Test

The cytotoxicity experiment validated the biosafety of peptoid samples through cell viability. Mouse fibroblast cells L929 were chosen as the experimental cells and cultured with a medium of RPMI-1640:FBS = 9:1 for resuscitation and passage. After 24 h of cell passage, the dead cells were removed by PBS washing. Trypsinization was performed, and the cells were centrifuged to remove the culture medium (and excess trypsin). The cell count was analyzed using a hemocytometer, and the cells were diluted to a concentration of 80,000 cells per milliliter of culture medium. Five columns were marked in the 96-well plate as 1, 5, 10, control, and blank, and three parallel experiments were conducted. A total of 100 μL of the cell suspension (approximately 8000 cells) was seeded into each experimental well. PBS buffer was added to the peripheral wells to prevent evaporation of the culture medium in the experimental wells. The cells in the plate were incubated in the cell incubator (37 °C, 5% CO_2_) for 24 h. Sample culture media at concentrations of 1, 5, and 10 mg·mL^−1^ were prepared and filtered into centrifuge tubes, which were then used to replace the culture media in columns 1, 5, and 10, respectively. After drug treatment, the cells were placed in the cell culture incubator for 24 h. The CCK-8 test reagent (culture medium: CCK-8 = 10:1) was then added to the cells in the dark, and the cells were incubated for an additional 2 h. The absorbance of cells in all experimental wells was measured using a plate reader. The relative cell viability was calculated using the formula, and the average value of the three parallel experiments was calculated:

$$\mathrm{Cell}\;\mathrm{Viability}\left(\%\right)=\left(\frac{A_{s}-A_{b}}{A_{c}-A_{b}}\right)\;\text{×}\;100\%$$

A_b_ is the absorbance value of the blank group containing only the medium, A_c_ is the absorbance value of the control group containing cells and the medium, and A_s_ is the absorbance value of the experimental group containing cells and the sample medium.

## 3. Results and Discussion

### 3.1. Design and Synthesis of Linear and Star-Shaped Peptoids (L-A_m_ and S-(A_n_)_3_)

Antifreeze proteins inhibit ice crystal growth by adsorbing onto the surface of ice crystals and altering the structure of the ice-water interface. Some studies showed that both hydrophilic and hydrophobic residues existed at the ice-binding sites of antifreeze proteins. Hydrophilic groups at the ice-binding sites form hydrogen bonds with water molecules on the surface of ice crystals. Hydrophobic groups interact with the ice-like structure on the ice crystal surface, causing the water molecules to arrange in a lattice-like manner. Additionally, hydrogen bonds anchor the water molecule lattice, ultimately enabling the binding of antifreeze proteins to ice crystals [28]. And this antifreeze mechanism has been found to occur in antifreeze proteins containing threonine residues. The presence of both hydrophilic (−OH) and hydrophobic (−CH_3_) groups in threonine made it a key structural characteristic that endowed antifreeze proteins with their excellent antifreeze properties [29]. Based on this, the isopropanol group was designed as the side-chain functional group of peptoids. It contained both a hydrophilic hydroxyl group and a hydrophobic methyl group. The solid-phase submonomer synthesis method was employed here to prepare peptoids. The side chains were introduced by using different primary amines in the substitution steps. Since isopropanolamine contained an alcohol hydroxyl group, the high reactivity of the active hydrogen could cause side reactions in the amine substitution reaction during the synthesis of the peptoid. Therefore, an organosilane protecting agent was used to selectively react with the hydroxyl group (Scheme S1) and then removed in the cleavage step. The successful protection of the hydroxyl group was confirmed by ^1^H NMR spectroscopy (Figure S1).

**Scheme 1:**
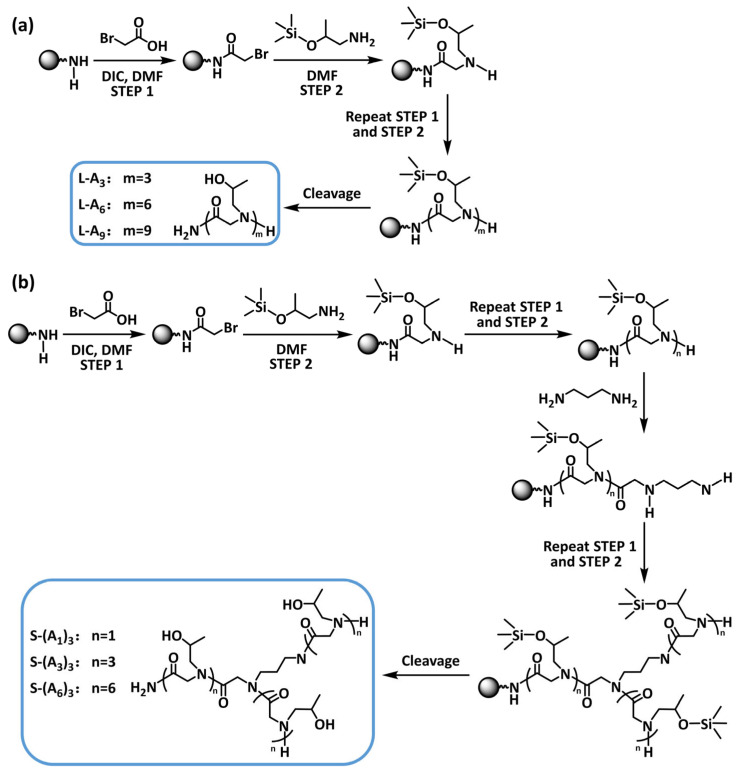
Molecular structures and synthetic routes of (**a**) linear and (**b**) star-shaped peptoids.

The linear (Scheme 1a) and star-shaped (Scheme 1b) peptoids were synthesized by the solid-phase submonomer synthesis method. The crude products obtained after cleavage with a TFA/H_2_O/TIS (*v*/*v*/*v* = 95/2.5/2.5) mixture were purified using preparative high-performance liquid chromatography. The molecular weight and purity of purified products were characterized by mass spectrometry (MS) (Figure S2) and high-performance liquid chromatography (HPLC) (Figure S3). The results proved that the molecular weights of all the purified peptoids were consistent with the designed structures. The main peak area in the HPLC spectra exceeded 90%, indicating that the purity of the synthesized peptoids was above 90%.

### 3.2. Ice Recrystallization Inhibition of Peptoids

**Figure 1:**
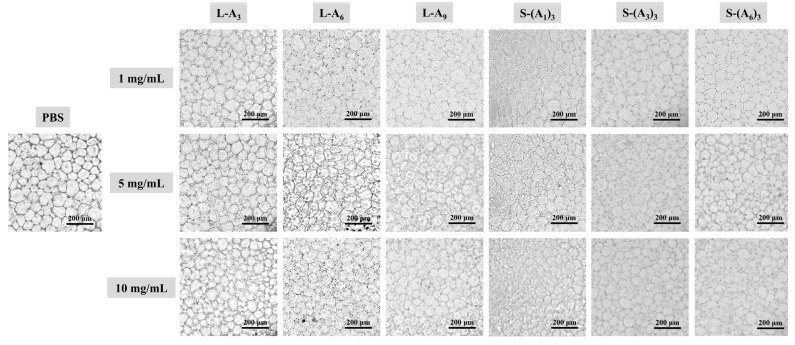
Microscopic images of ice crystals in solutions of linear and star-shaped peptoids compared to PBS solution (after annealing at −6 °C for 30 min).

**Figure 2:**
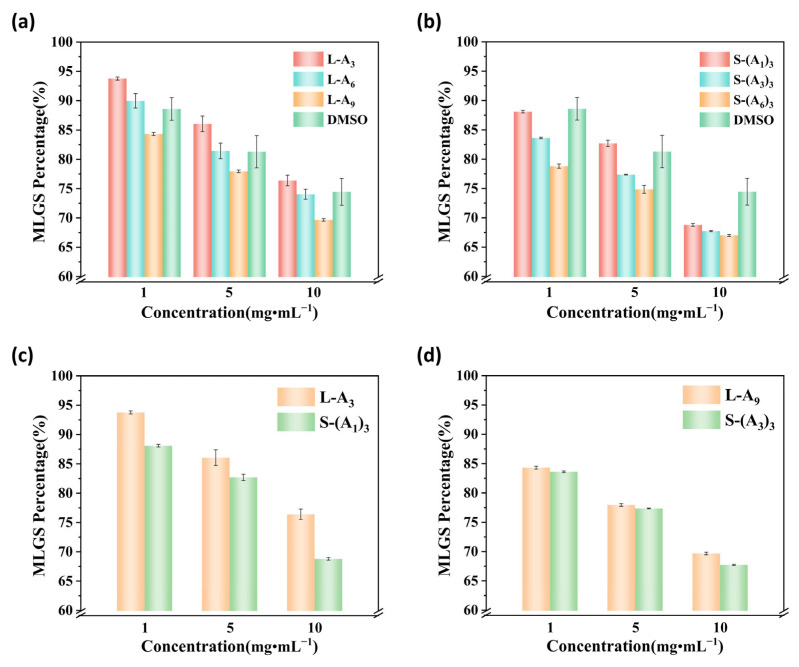
MLGS for (**a**) linear and (**b**) star-shaped peptoid solutions with different chain lengths compared to DMSO (as a percentage of PBS solution MLGS); (**c**,**d**) comparison of MLGS for two sets of linear and star-shaped peptoid solutions with the same chain length (as a percentage of PBS solution MLGS).

Ice recrystallization during cell resuscitation can form larger ice crystals, causing cell damage. The ice recrystallization inhibition (IRI) activity is an important indicator for evaluating the protective efficiency of cryoprotective agents. The mean largest grain size (MLGS) of ice crystals in PBS solutions containing different concentrations (1 mg·mL^−1^, 5 mg·mL^−1^, 10 mg·mL^−1^) of peptoids after annealing at −6 °C for 30 min was measured using a Linkam cryostage (C194) to assess the IRI activity of peptoids. The images in Figure 1 showed that within the same field of view, the PBS solution had fewer and larger ice crystals. After the addition of peptoids, the ice crystal sizes were significantly reduced, and the number of small ice crystals increased. Moreover, as the peptoid concentration increased, the distribution of ice crystals became more uniform. This indicated that all peptoids have an inhibitory effect on ice recrystallization, regardless of peptoid chain length or topological structure. The MLGS of PBS was 163.16 μm, while the MLGS of the star-shaped peptoid S-(A_6_)_3_ solution at a concentration of 10 mg·mL^−1^ was only 110.22 μm. With the increase in peptoid concentration and chain length, the IRI activity increased, and the same trend was observed for both linear and star-shaped peptoids (Figure 2a,b). When the concentration increased from 1 mg·mL^−1^ to 10 mg·mL^−1^, the MLGS percentage of L-A_3_ and S-(A_1_)_3_ decreased from 93.8% and 88.1% to 76.4% and 68.8%, respectively. When the degree of polymerization (chain length) enhanced from 3 to 9, the MLGS percentage dropped from 76.4% (L-A_3_) and 68.8% (S-(A_1_)_3_) to 69.7% (L-A_9_) and 67.7% (S-(A_3_)_3_), respectively. Moreover, the MLGS of L-A_9_, S-(A_3_)_3_, and S-(A_6_)_3_ peptoid solutions were found to be lower than that of DMSO solutions at all concentrations. This demonstrated that the synthesized long-chain peptoids had higher IRI activity than DMSO. The linear and star-shaped peptoids with the same degree of polymerization, i.e., the same amount of isopropanol side chains, were compared with the MLGS to study the topology effect on IRI activity. For example, S-(A_1_)_3_ and L-A_3_ both have three repeating units (three isopropanol side chains), and the MLGS of their solutions were compared. It is shown in Figure 2c,d that the MLGS of star-shaped peptoids is smaller than that of linear ones. These findings suggested that peptoids with longer chain lengths and star shapes have better IRI activity. The reason could be that these peptoids matched the ice crystal lattice better, thus inhibiting ice recrystallization more.

### 3.3. Ice Growth Inhibition of Peptoids

**Figure 3:**
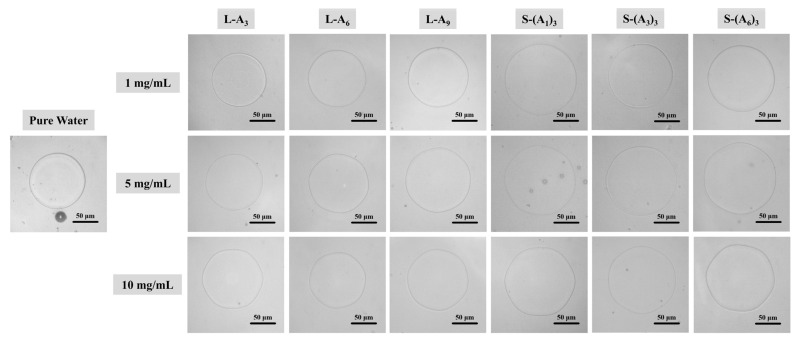
Microscopic images of individual ice crystal morphologies for linear and star-shaped peptoid solutions compared to pure water (ΔT = 0.10 °C).

To evaluate the effect of peptoids on the morphology and growth rate of ice crystals, a nanoliter osmometer was used to adjust supercooling temperature, and a high-speed camera was employed to record the growth process of individual ice crystals in peptoid solutions. The morphologies of individual ice crystals in pure water and peptoid solutions of varying concentrations at a supercooling temperature of 0.10 °C (Figure 3) were observed. The ice crystals in pure water appeared round-shaped. In contrast, ice crystals in all peptoid solutions grew towards a hexagonal shape. This morphological behavior indicated that peptoids can adsorb to the surface of ice crystals through hydrogen bonds and hydrophobic interactions, thus regulating ice shape like AFPs. For both linear and star-shaped peptoids, as the concentration and polymerization degree of the peptoids increased, the interactions between peptoids and ice became stronger, and more pronounced hexagonal shapes of the ice crystals were observed. Similarly, at other different supercooling temperatures (ΔT = 0.02, 0.04, 0.06, 0.08 °C), all ice crystals of peptoid solutions grew into hexagonal shapes. As the supercooling temperature increased, the growth rate of ice crystals increased. Peptoid molecules covered the surface of the ice crystals, inhibiting their growth, while the uncovered regions grew more rapidly. As a result, the hexagonal morphology of the ice crystals became more pronounced.

**Figure 4:**
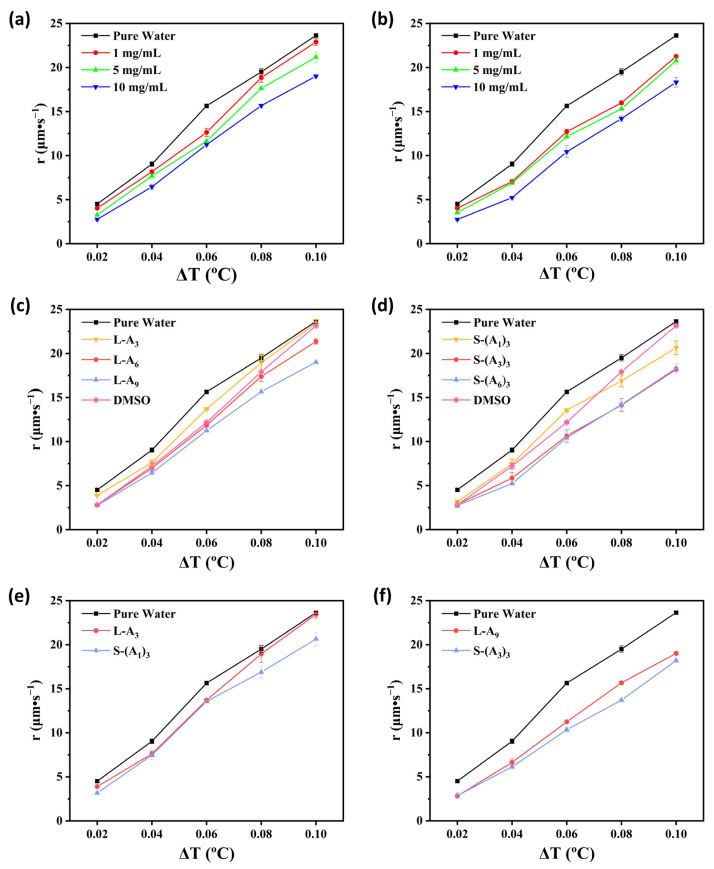
Comparison of ice crystal growth rates at various supercooling temperatures for (**a**) L-A_9_ and (**b**) S-(A_6_)_3_ peptoid solutions at different concentrations with pure water; (**c**) linear and (**d**) star-shaped peptoid solutions (10 mg·mL^−1^) compared to pure water and DMSO solution (10 mg·mL^−1^); (**e**,**f**) two sets of linear and star-shaped peptoid solutions with equivalent chain lengths (10 mg·mL^−1^) compared to pure water.

Obviously, as the supercooling temperature increased, the growth rate of ice crystals in both pure water and peptoid solutions became faster (Figure 4 and Figure S4). The growth rates of ice crystals in linear peptoid L-A_9_ solutions with concentrations of 1 mg·mL^−1^, 5 mg·mL^−1^, and 10 mg·mL^−1^ at a supercooling temperature of 0.10 °C are 22.89 μm·s^−1^, 21.16 μm·s^−1^, and 19.01 μm·s^−1^ (Figure 4a), respectively. While under the same conditions, the growth rates of ice crystals in star-shaped peptoid S-(A_6_)_3_ solutions are 21.27 μm·s^−1^, 20.78 μm·s^−1^, and 18.86 μm·s^−1^ (Figure 4b), respectively. As the concentration of peptoid increased, the growth rate of ice crystals decreased at all tested supercooling temperatures, indicating a stronger inhibition effect on ice crystal growth. This result was consistent with the conclusions of previous studies by our research group [24,25]. It is worth emphasizing that in Figure 4c,d, for both linear and star-shaped peptoids, the longer the chain length, the slower the ice crystal growth rate was, indicating a stronger ability to inhibit ice crystal growth. Moreover, the ice crystal growth rate of long-chain peptoid solutions was significantly lower than that of the DMSO solution (Figure 4c,d and Figure S5), demonstrating that the peptoids had a much greater ability to inhibit ice crystal growth than DMSO. This is because more hydrophilic and hydrophobic groups increase the binding sites for hydrogen bonds and hydrophobic interactions, which enhances the ability to inhibit ice crystal growth. However, in comparison to the S-(A_3_)_3_, the growth rate of ice crystals in the 10 mg·mL^−1^ S-(A_6_)_3_ peptoid solution did not significantly decrease. This suggests that an increase in peptoid chain length does not endlessly enhance the ability to inhibit ice crystal growth. To study the effect of topology on ice crystal growth inhibition, linear and star-shaped peptoids with the same polymerization degree (the same amount of side chains) were compared (Figure 4e,f). Especially at higher supercooling temperatures, the ice crystal growth rates of star-shaped peptoid S-(A_1_)_3_ and S-(A_3_)_3_ solutions at a concentration of 10 mg·mL^−1^ were lower than those of linear peptoid L-A_3_ and L-A_9_ solutions, respectively. The results indicate that star-shaped peptoids have a stronger ability to inhibit ice crystal growth. This is likely because their topology can enhance control over the ice-water interface, thus further suppressing the growth of ice crystals. These results agree with the IRI study that longer peptoid chain length and star shape are preferred for better antifreeze properties.

### 3.4. The Cytotoxicity of Peptoids

**Figure 5:**
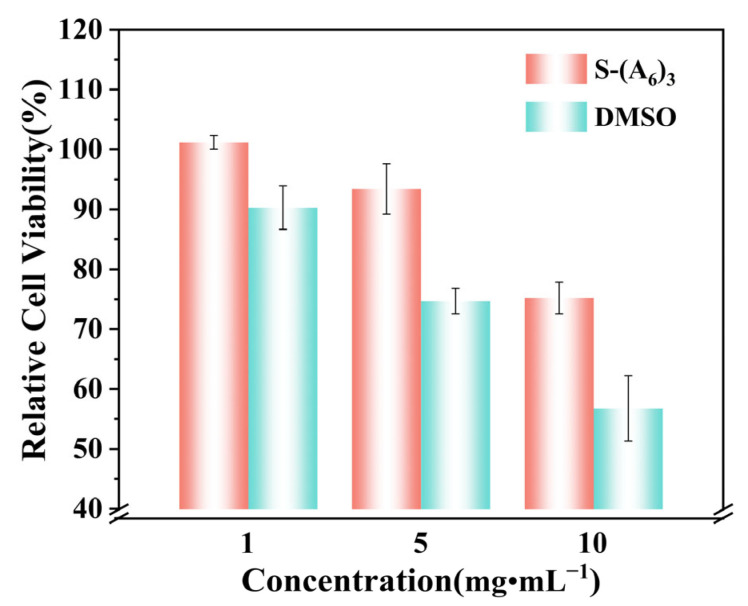
Relative cell viability in culture media containing different concentrations of star-shaped peptoid S-(A_6_)_3_ and DMSO.

Based on the antifreeze experimental results, it was found that the star-shaped long-chain peptoid S-(A_6_)_3_ exhibited the best IRI activity and inhibition of ice crystal growth. Therefore, cytotoxicity tests were conducted to evaluate the biosafety of S-(A_6_)_3_ for cryopreservation. If the cell viability is ≥75%, it is generally considered that the tested sample is non-toxic to cells [30]. The relative cell viability in culture media containing star-shaped peptoid S-(A_6_)_3_ at concentrations of 1 mg·mL^−1^, 5 mg·mL^−1^, and 10 mg·mL^−1^ all reached over 75% (Figure 5), suggesting that S-(A_6_)_3_ was non-cytotoxic up to 10 mg·mL^−1^. Compared with the commonly used cryoprotectant DMSO, the relative cell viability in culture media with S-(A_6_)_3_ at all experimental concentrations was higher. The relative cell viability of cells cultured with 10 mg·mL^−1^ DMSO was only 55%, which will cause severe damage to cells. Therefore, star-shaped peptoids are highly likely to become an alternative to DMSO as a novel, efficient, and non-toxic cryoprotectant.

## 4. Conclusions

In summary, a series of linear and star-shaped peptoids mimicking antifreeze proteins were successfully synthesized, and their antifreeze properties were evaluated. A structure-property relationship study revealed that longer chain length of peptoids enhanced both ice recrystallization inhibition and ice growth suppression. Furthermore, star-shaped peptoids outperformed their linear counterparts, likely due to their improved interaction with ice interfaces. The best-performing peptoid, S-(A_6_)_3_, not only exhibited superior antifreeze properties compared to DMSO but also demonstrated good biosafety. It can be used as a promising, efficient, and non-toxic cryoprotectant. This study offers a new direction for the rational design of next-generation cryoprotectants with potential applications in cryobiology and biomedicine.

## Data Availability

The data that support the findings of this study are available from the corresponding author upon request.

## Supplementary Materials

The following supporting information can be downloaded at: https://www.mdpi.com/article/10.3390/biomimetics10060368/s1, Scheme S1: Protection route of the hydroxyl group in isopropanolamine; Figure S1: ^1^H NMR spectrum of isopropanolamine submonomer after protection in CDCl_3_; Figure S2: MS spectra of linear and star-shaped peptoids after purification. (a) L-A_3_; (b) L-A_6_; (c) L-A_9_; (d) S-(A_1_)_3_; (e) S-(A_3_)_3_; (f) S-(A_6_)_3_; Figure S3: HPLC spectra of linear and star-shaped peptoids after purification. (a) L-A_3_; (b) L-A_6_; (c) L-A_9_; (d) S-(A_1_)_3_; (e) S-(A_3_)_3_; (f) S-(A_6_)_3_; Figure S4: Comparison of ice crystal growth rates at various supercooling temperatures. (a) L-A_3_; (b) L-A_6_; (c) L-A_9_; (d) S-(A_1_)_3_; (e) S-(A_3_)_3_; (f) S-(A_6_)_3_; Figure S5: Ice crystal growth rate of DMSO at various supercooling temperatures.
