# Supplementary material for: Topology-Dependent Antifreeze Properties of Biomimetic Linear and Star-Shaped Peptoids
Source: Biomimetics (Basel). 2025 Jun 4;10(6):368. doi: 10.3390/biomimetics10060368 (PMC12190692; doi:10.3390/biomimetics10060368)
Supplement: Supplementary file 1 [file biomimetics-10-00368-s001.zip › biomimetics-3629370-supplementary.pdf]

Supplementary Materials

# Topology-Dependent Antifreeze Properties of Biomimetic Linear and Star-Shaped Peptoids

Lei Feng,[1] Liugen Xu,[1] Junhao Wen,[1] Minghai Zhao,[1] Amjad Ali,[2] Naushad Ahmad,[3] Jianwei Lu[1,*] and Li Guo[1,*]

[1]*School of Materials Science and Engineering, Jiangsu University, Zhenjiang, 212013, P.R.China*

[2]*College of Materials Science and Engineering, Nanjing Forestry University, Nanjing, 210037, P.R.China*

[3]*Department of Chemistry, College of Science, King Saud University, Riyadh-11451, Saudi Arabia*

*Correspondence to: Li Guo (Email: liguo@ujs.edu.cn)*

*Jianwei Lu (Email: jianwei@ujs.edu.cn)*

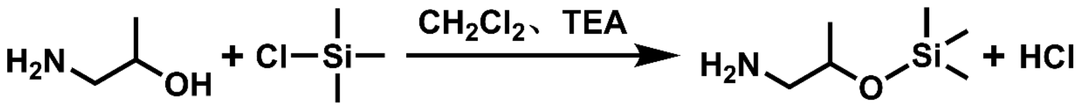


**Scheme S1.** Protection route of the hydroxyl group in isopropanolamine.

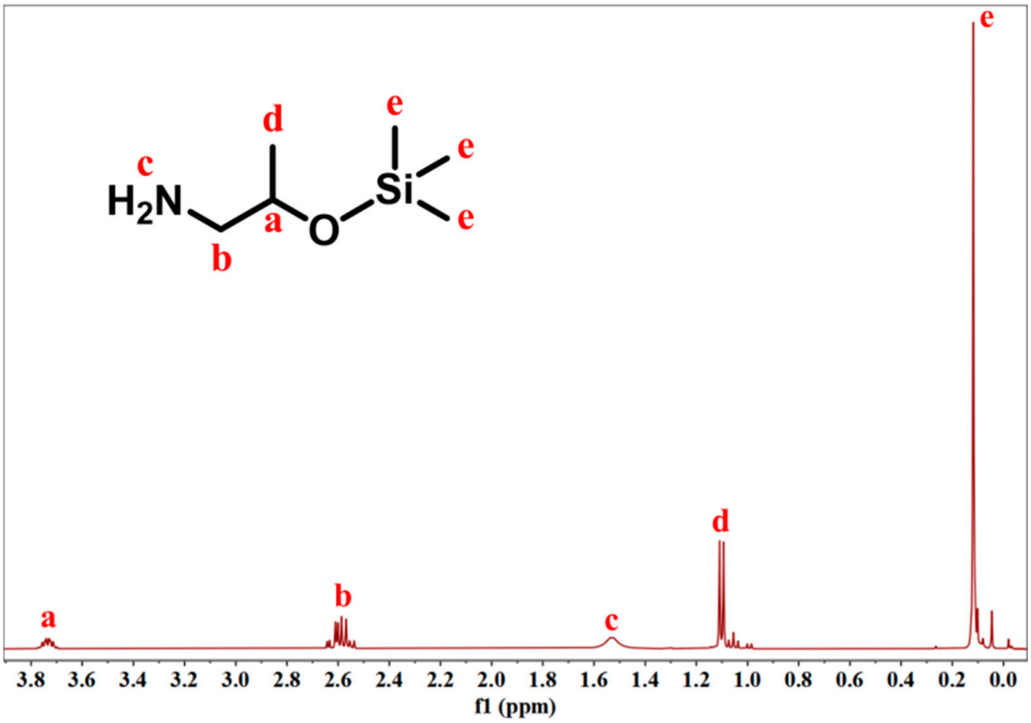


**Figure S1.** $^1H$ NMR spectrum of isopropanolamine submonomer after protection in $CDCl_3$.

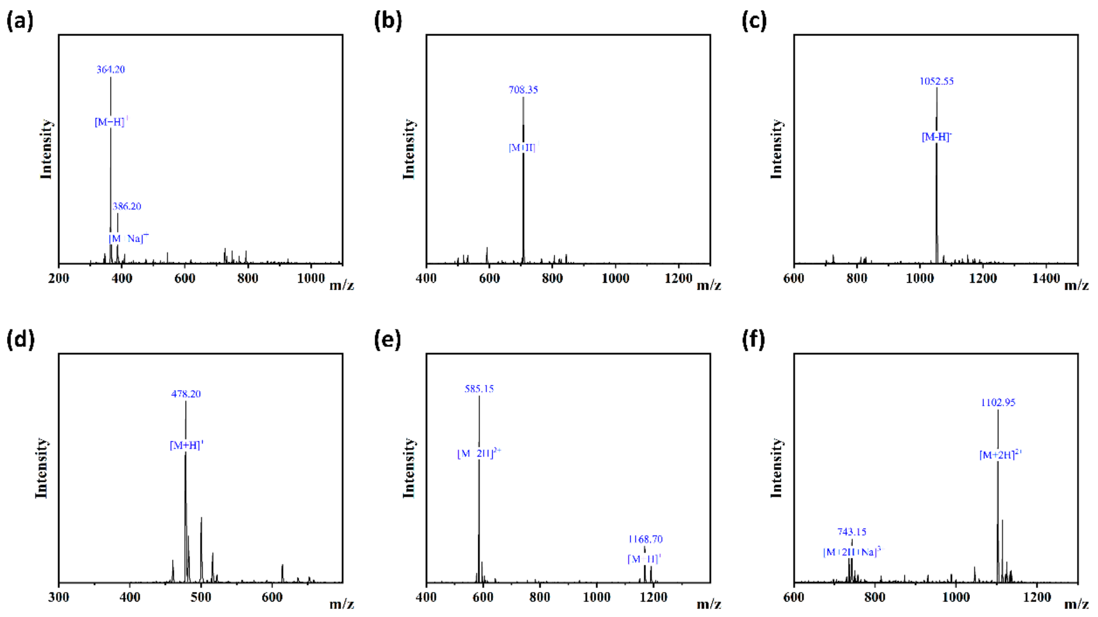


**Figure S2.** MS spectra of linear and star-shaped peptoids after purification. (a) L-A3; (b) L-A6; (c) L-A9; (d) S-(A1)3; (e) S-(A3)3; (f) S-(A6)3.

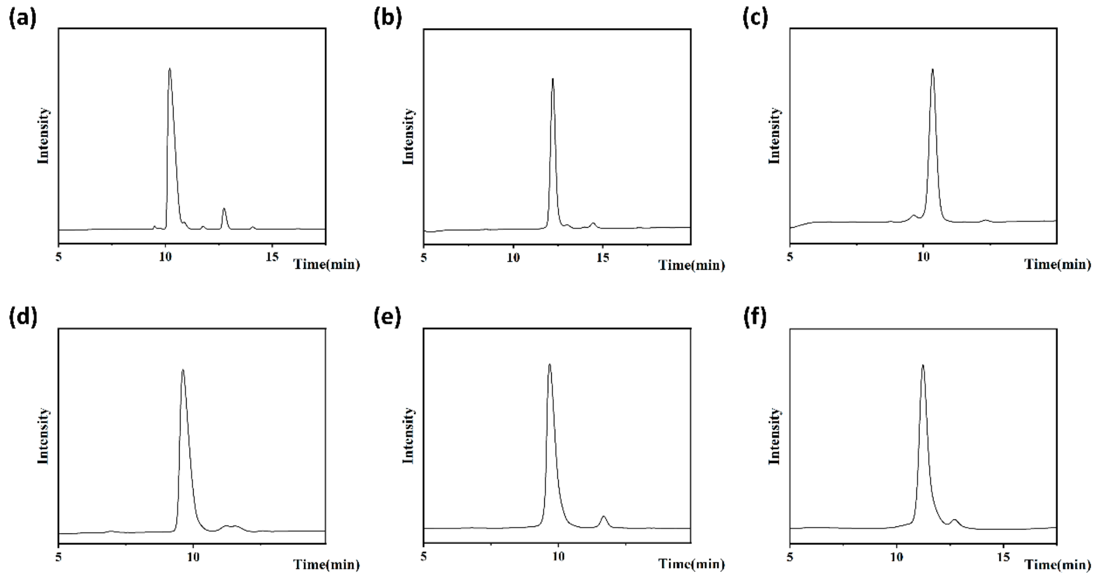


**Figure S3.** HPLC spectra of linear and star-shaped peptoids after purification. (a) L-A3; (b) L-A6; (c) L-A9; (d) S-(A1)3; (e) S-(A3)3; (f) S-(A6)3.

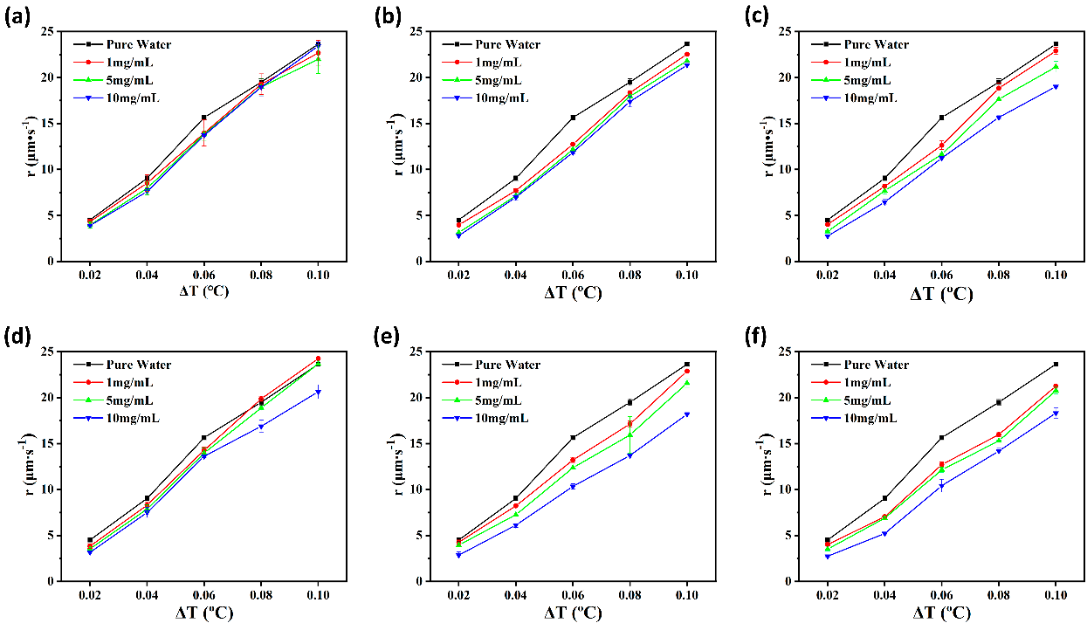


**Figure S4.** Comparison of ice crystal growth rates at various supercooling temperatures. (a) L-$A_3$; (b) L-$A_6$; (c) L-$A_9$; (d) S-$(A_1)_3$; (e) S-$(A_3)_3$; (f) S-$(A_6)_3$.

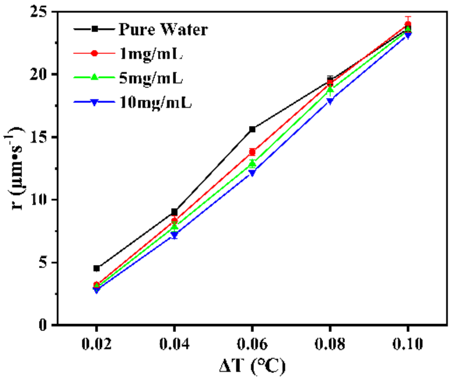


**Figure S5.** Ice crystal growth rate of DMSO at various supercooling temperatures.
